# A Comparative Study of Three Types Shear Mode Piezoelectric Wafers in Shear Horizontal Wave Generation and Reception

**DOI:** 10.3390/s18082681

**Published:** 2018-08-15

**Authors:** Qiang Huan, Mingtong Chen, Faxin Li

**Affiliations:** 1 LTCS and College of Engineering, Peking University, Beijing 100871, China; qiang_huan@pku.edu.cn (Q.H.); chenmingtong@pku.edu.cn (M.C.); 2 Center for Applied Physics and Technology, Peking University, Beijing 100871, China; 3 Beijing Key Laboratory of Magnetoelectric Materials and Devices, Peking University, Beijing 100871, China

**Keywords:** guided wave, shear horizontal wave, piezoelectric transducers, thickness shear, face shear

## Abstract

Guided wave-based inspection has emerged as a promising tool to evaluate the reliability of key components in modern industries. The fundamental shear horizontal (SH_0_) wave is always of great interests for plate-like structures because of its non-dispersion characteristics. However, the generation and reception of SH_0_ wave using piezoelectric wafers is not straightforward. In this paper, we firstly define three types shear mode piezoelectric wafers, i.e., the conventional in-plane poled thickness-shear (d_15_) mode, the thickness-poled thickness-shear (d_15_) mode, and the face-shear (d_24_) mode. Then, finite element simulations were conducted to demonstrate their performance in SH wave generation and reception. The results indicated that the face shear d_24_ wafer can generate almost single mode SH_0_ wave, while both types of d_15_ wafers would generate Lamb waves and SH_0_ wave simultaneously. Finally, experiments were carried out to check the efficiency of different shear mode piezoelectric wafers in SH_0_ wave generation and reception. The results indicated that the d_24_ wafer can generate and receive SH_0_ wave of high signal to noise ratio (SNR) with high energy conversion efficiency, while the in-plane poled d_15_ wafer would generate SH_0_ wave of high amplitude and acceptable SNR but with relatively low energy conversion efficiency. The performances of thickness-poled d_15_ wafer was not as good as the other two in both SH wave generation and reception. This work will be helpful for the applications of SH waves in plate-like structures.

## 1. Introduction

Nondestructive testing (NDT) and structural health monitoring (SHM) are of great importance in modern industries to improve the reliability of structures and components [1,2]. Guided wave- based techniques have attracted special attention for their rapid and wide range of inspection applications [3,4,5]. For plate-like structures, there exist two types of guided waves: Lamb waves and shear horizontal (SH) waves. In the past two decades, Lamb wave-based inspections had been extensively studied and applied due to the easy excitation and reception by thickness-poled piezoelectric (PZT) wafers [6,7,8,9,10,11,12,13,14]. However, the inherent multi-modal and dispersive characteristics of Lamb waves have seriously restricted their further development. 

In comparison, the fundamental shear horizontal (SH_0_) wave in plates is completely non-dispersive. Because of its uncoupled displacements with Lamb waves, in theory it is possible to generate single mode SH_0_ wave. Besides, it exhibits some other attractive feathers such as less mode conversion and low attenuation in fluid-loaded plates [15,16,17]. Unfortunately, the generation and reception of SH_0_ wave is not as convenient as that of Lamb waves. Although in the late 1970s, Thompson had successfully excited SH_0_ wave in metal plates by using electromagnetic acoustic transducers (EMATs) [18,19], SH wave-based inspection has not been widely used yet because of the rather low efficiency of EMATs. Actually, SH waves can be excited by using the conventional in-plane poled thickness-shear (d_15_) piezoelectric wafers [20]. However, Lamb waves will be excited simultaneously [21]. The conventional d_15_ wafers can also be assembled to be a ring or a planar array to act as the fundamental torsional (T(0,1) ) wave transducers in pipes [22,23,24] or omnidirectional SH wave transducers in plates [25]. Recently, another two types of shear mode piezoelectric wafers have been developed in our group for generation/reception of SH waves, i.e., the face-shear d_24_ wafer and the thickness-poled d_15_ wafer [26,27,28]. The d_24_ wafer can excite single-mode SH wave and receive SH wave only by filtering Lamb waves [26]. It can also form a ring to generate single-mode T(0,1) [29] wave in pipes and form a circular array to generate SH wave omni-directionally in plates [30]. The advantage of the thickness-poled d_15_ wafer is that it can easily form a uniform-sensitivity omni-directional SH wave transducer based on the thickness-poled PZT ring [27,28].

Now we can see that all the above mentioned three types of shear mode piezoelectric wafers can be used to generate/receive SH waves in plates and torsional waves in pipes. However, the efficiencies of these shear mode piezoelectric wafers were not compared yet. Thus, in this work, we conducted a comparative study on the performances of these three types of shear mode piezoelectric wafers in excitation and reception of SH waves in plates. Firstly, the definitions of these three types of shear mode piezoelectric wafers were presented. Then the wave patterns generated by different shear mode piezoelectric wafers were investigated by using finite element simulations. Finally, experiments were carried out to examine the efficiency of different shear mode piezoelectric wafers in SH_0_ wave generation and reception.

## 2. Definition of Three Types Shear Modes in Piezoelectric Wafers 

**Figure 1:**
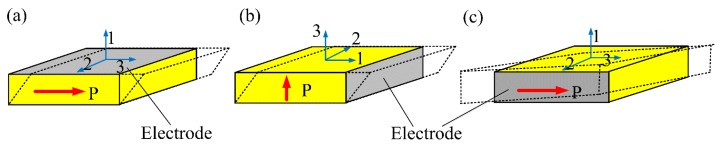
Definition of different shear mode piezoelectric wafers: (**a**) conventional in-plane poled thickness-shear d_15_ mode; (**b**) thickness-poled thickness-shear d_15_ mode; (**c**) face shear d_24_ mode.

When we talk about the shear mode in piezoelectric ceramics, we usually denote the conventional thickness-shear (d_15_) mode. That is, a conventional d_15_ mode piezoelectric wafer is in-plane poled and the electric field is applied along the thickness direction, as shown in Figure 1a. Actually, for wafer-shaped piezoelectric ceramics used for guided wave generation/reception, there also exist another two shear modes, i.e., the thickness-poled thickness-shear (d_15_) mode as shown in Figure 1b and the in-plane poled face-shear (d_24_) mode as shown in Figure 1c. From the material point of view, these three types of shear modes are equivalent since in all of them the electric field is applied perpendicularly to the poling direction. However, for wafer-shaped samples, they are different. Note that the thickness-poled d_15_ mode and the face-shear d_24_ mode have special advantages when used for SH wave excitation/reception [26,27].

**Figure 2:**
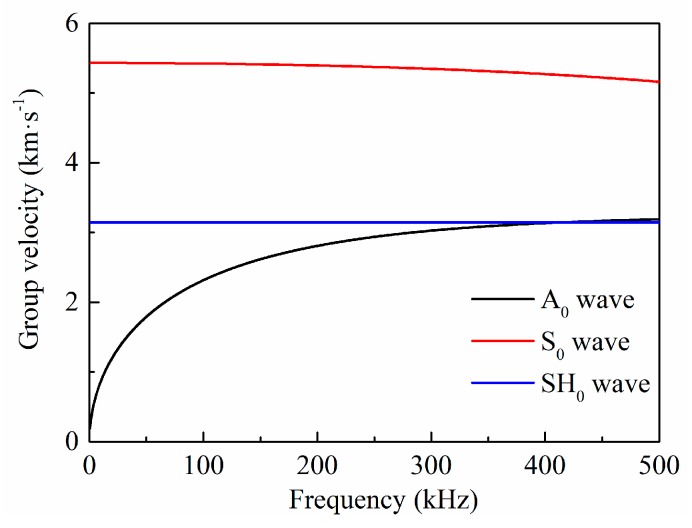
Group velocity verse frequency for SH_0_ wave and Lamb waves in a 2 mm-thick aluminum plate.

In order for the convenience of analysis, Figure 2 presented the group velocity of guided waves for a 2 mm-thick aluminum plate. It can be found that before the cut-off frequency, there only exist three wave modes, i.e., the A_0_ wave, S_0_ wave and SH_0_ wave. However, both the S_0_ wave and A_0_ wave are dispersive, which would lead signal distortion after wave propagation since the signal is excited in a certain bandwidth. In comparison, SH_0_ wave is totally non-dispersive.

## 3. Finite Element Simulations 

Time-transient finite element (FEM) simulations were firstly performed using ANSYS to investigate the wave radiation patterns generated by different shear mode piezoelectric wafers. The dimensions of the three types shear mode piezoelectric wafers used here were all 8 mm × 8 mm × 1 mm. The material was PZT-5H whose parameters can be found in [31].

**Figure 3:**
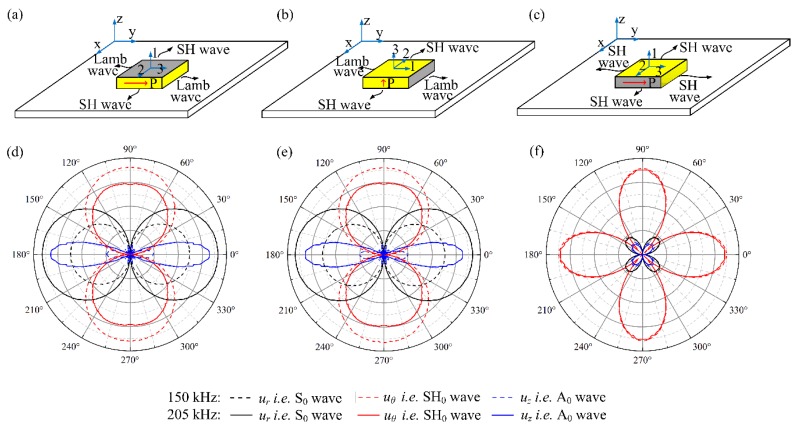
Illustration (up) and simulated results (bottom) of wave radiation patterns generated by using different shear modes piezoelectric wafers at 150 kHz and 205 kHz. (**a**,**d**): in-plane poled d_15_ mode; (**b**,**e**): thickness-poled d_15_ mode; (**c**,**f**): face shear d_24_ mode.

The waveguide was selected to be a 400 mm × 400 mm × 2 mm aluminum plate with the PZT wafer bonded on the center of it. The largest size of elements is set to be less than 1/20 the shortest wavelength, and the time step was set to be less than 1/20 of the central frequency. Figure 3 presented the FEM simulated results of wave patterns generated by the three types shear mode piezoelectric wafers at 150 kHz and 205 kHz. In cylindrical coordinates, the radial displacement component *u_r_*, tangential displacement component *u_θ_*, and out-of-plane displacements component *u_z_* are dominated by the S_0_ wave, SH_0_ wave, and A_0_ wave, respectively [32]. Hence, we can verify that *u_r_*, *u_θ_*, and *u_z_* were associated with the S_0_ wave, SH_0_ wave and A_0_ wave, respectively. It can be seen from Figure 3a,b,d,e that both the in-plane poled and thickness-poled d_15_ piezoelectric wafers can generate SH_0_ wave along the bi-directions (90° and 270°) perpendicular to both the poling direction and the field direction, and simultaneously generate Lamb waves along the orthogonal in-plane directions (0° and 180°). At multiple frequencies, for both d_15_ mode wafers, the maximum amplitudes of the generated Lamb waves were always comparable to that of the SH_0_ wave. The decreased amplitude of the A_0_ wave at 150 kHz was mainly caused by the effect of turning frequency [33]. Moreover, the radiation angle of the generated A_0_ wave is always significantly smaller than that of the generated S_0_ wave and SH_0_ wave. These results were in agreement with those reported in [21]. Note that the wave radiation patterns generated by using these two types d_15_ piezoelectric wafers are exactly the same since they are both thickness shear modes. 

In comparison, the generated wave pattern by the face shear d_24_ piezoelectric wafer was quite different, as shown in Figure 3c,f. The SH_0_ wave was generated along four main directions (0°, 90°, 180° and 270°) with four-fold rotational symmetry. When deviating from the main directions, its amplitude decreased quickly and vanished at the wafer’s diagonals (45°, 135°, 225° and 315°) where Lamb waves including the A_0_ wave and S_0_ wave were generated with much smaller amplitudes. This phenomenon is not difficult to understand. Because of the pure face-shear deformation of the d_24_ wafer, shear stress would be generated along the four sides of the wafer with the same amplitude, resulting in the generated four-fold rotational symmetric SH_0_ wave. Meanwhile, the shear stress would synthesize tensile/compressive stresses along the wafer’s diagonals, leading to the generation of Lamb waves. It should be noted that at multiple frequencies, the wave field generated by the d_24_ wafer was always dominated by the SH_0_ wave. These results were in good agreement with those reported in [26].

## 4. Experiments 

**Figure 4:**
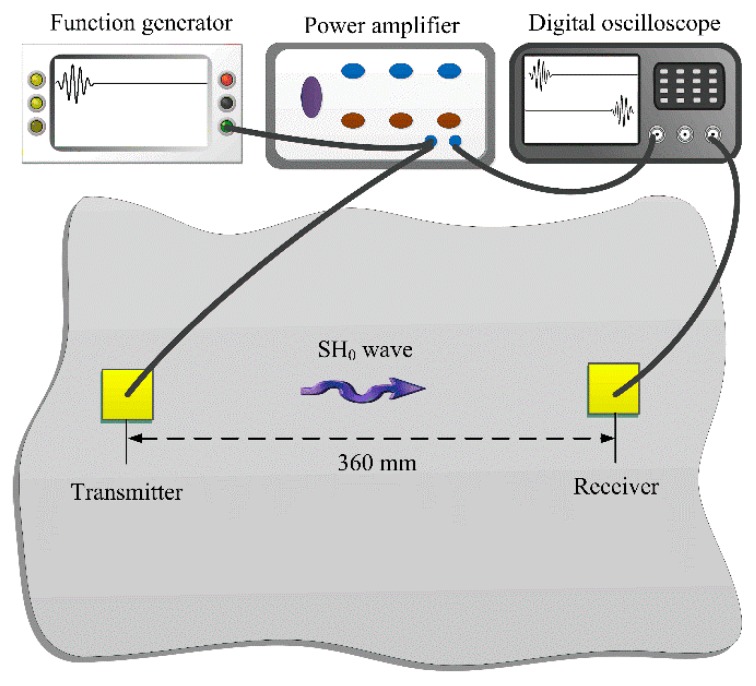
The schematic of experimental setup.

Experiments were then carried out to examine the efficiencies of different shear mode piezoelectric wafers in SH_0_ wave generation and reception. The experimental setup was shown in Figure 4.

A 1000 mm × 1000 mm × 2 mm aluminum plate was used as the waveguide. The size of all three types of piezoelectric wafers was 8 mm × 8 mm × 1 mm. The distance between the actuator and sensor was fixed at 360 mm so that the wave package can be separated in the time domain. When checking the wave generation performance, the different shear mode piezoelectric wafers served as actuators and the d_36_ type PMN-PT wafer (5 mm × 5 mm × 1 mm) was used as a sensor. Since the d_36_ type PMN-PT wafer can generate and receive both SH_0_ wave and Lamb waves, the wave velocity and purity can be examined at the same time [32]. When examining their performances in wave reception, the d_36_ type PMN-PT wafer served as the actuator and the piezoelectric wafers were used as sensors. Finally, the same shear mode piezoelectric wafers were used as both actuator and sensor to further investigate its performance. During testing, a five-cycle sinusoid tone-burst modulated into the Hanning window was used as the exciting signal. The signal was generated by a function generator (3320A, Agilent, Palo Alto, CA, USA) and amplified by a power amplifier (Model 7602M, KROHN-HITE, Brockton, MA, USA). An oscilloscope (Agilent DSO-X 3024A) was used to record the signals received by the sensors with a tracing average of 128 times. 

**Figure 5:**
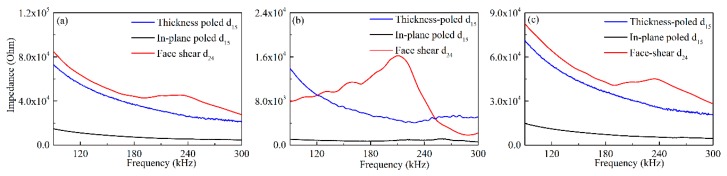
The impedance spectrum of three types shear mode piezoelectric wafers with the same dimensions of 8 mm × 8 mm × 1 mm bonded on the aluminum plate: (**a**) impedance; (**b**) resistance; (**c**) reactance.

Before formal testing, the impedance of these three types shear mode piezoelectric wafers bonded on an aluminum plate were measured by using an impedance analyzer (Agilent 4294A), and the results from 90 kHz to 300 kHz were shown in Figure 5. It can be seen in Figure 5a that the impedances (including resistance and reactance) of the face-shear d_24_ mode wafer and the thickness-poled d_15_ mode were close to each other at most frequencies, and both are much larger than that of the conventional in-plane poled d_15_ wafer. This is easy to understand because the in-plane poled d_15_ wafer has larger electrodes and smaller distances between the electrodes. Therefore, it is expected that the normal drive voltage of the in-plane poled d_15_ wafer should be lower than the other two. The non-monotonic of the impedance for the d_24_ wafer near 200 kHz was because there exists a resonance peak. Figure 5b,c presented the real image part i.e., resistance and imaginary part i.e., reactance of the impedance respectively. As shown in Figure 5b, for both two types d_15_ wafer, the resistance decreased firstly and then increased slightly with the increasing frequency. However, the changes in amplitude for the in-plane poled d_15_ wafer was much smaller than that of thickness-poled d_15_ wafer. In comparison, the resistance of the d_24_ wafer increased firstly and then decreased quickly with the increasing frequency. The inflection point of its curves was near its resonance frequency i.e., 200 kHz. Regarding the reactance shown in Figure 5c, for both two types d_15_ wafer, it decreased continuously with frequency increasing. One thing should be noted that for both two types d_15_ wafer, the amplitudes’ change in reactance was much larger than that of resistance, resulting in continue decreasing of their impedance with the increasing frequency, as shown in Figure 5a. For the reactance of the d_24_ wafer, there appeared slight increase in a small frequency range near 200 kHz. The changes in the resistance and reactance for the d_24_ wafer near 200 kHz codetermined its non-monotonic in impedance. It should be noted that the impedance of the wafers was dependent on their size, material and deformation mode.

### 4.1. Performance of Different Shear Mode Piezoelectric Wafers in SH Wave Generation 

Firstly, the performance of different shear mode piezoelectric wafers in SH_0_ wave generation was compared. The signals were generated by different shear mode wafers and received by the d_36_ type PMN-PT wafer. For all three types of wafer of 8 mm × 8 mm × 1 mm size, according to our previous work, the SH_0_ wave can be effectively generated from 90 kHz to 270 kHz in the 2 mm-thick aluminum plate. Hence the testing frequency was set in this range. During experiments, all wafers were excited by using a fixed voltage of 20 V firstly and then using a fixed power consumption of 0.08 W.

**Figure 6:**
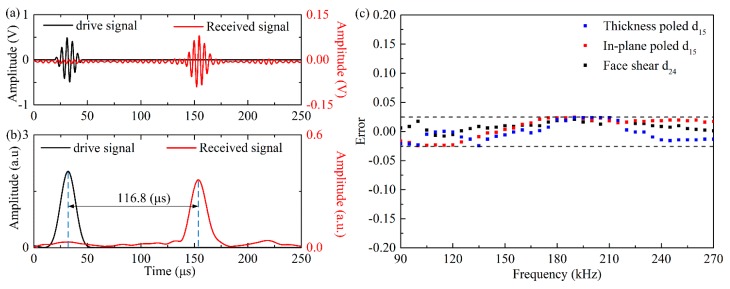
(**a**) Wave signals generated by the d_24_ piezoelectric wafer and received by the d_36_ type PMN-PT wafer with drive voltage of 20 V at 200 kHz; (**b**) continue wavelet transform (CWT) of the drive signal and receive signal in (**a**); (**c**) Frequency dependent relative errors of the measured group velocity of the SH_0_ waves generated by different shear mode piezoelectric wafers.

**Figure 7:**
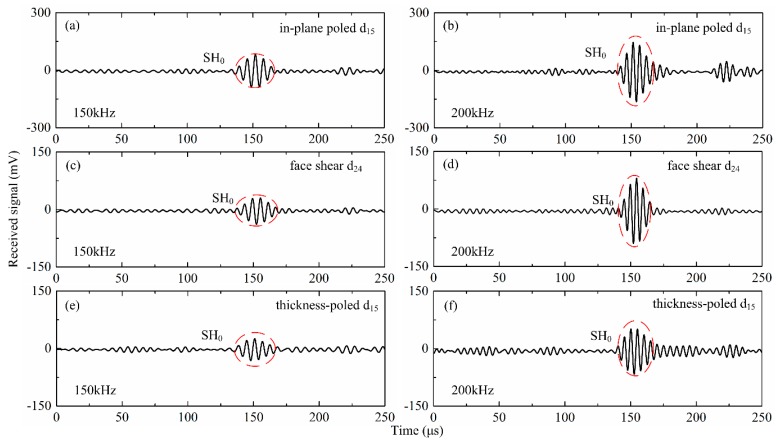
Wave signals generated by different shear mode piezoelectric wafers and received by the d_36_ type PMN-PT wafer with drive voltage of 20 V at 150 kHz (left) and 200kHz (right). (**a**,**b**): in-plane poled d_15_ mode; (**c**,**d**): face shear d_24_ mode; (**e**,**f**): thickness-poled d_15_ mode.

In order to verify the wave velocity generated by the different types wafers, the continuous wavelet transform (CWT) was adopted to analyze the signals. Due to the space limitations, only the wave generated by the d_24_ wafer with drive voltage of 20 V at 200 kHz was presented as an example. It can be seen in Figure 6a that only one wave package appeared in the received signal. After applying the CWT to the signals, the time internal between the drive signal and the received signal was recognized to be 116.8 μs, as shown in Figure 6b. The corresponding group wave velocity was calculated to be 3082 mm·s^−1^, which was agreement with the theoretical value of SH_0_ wave in an aluminum plate, i.e., 3099 mm·s^−1^. The group velocity of the waves generated by different shear mode piezoelectric wafers verses frequency was plotted in Figure 6c. It can be found that within the testing frequency range, the experimental results for all three types wafers was agreement with theoretical value of SH_0_ wave within error less than 2.5%. Figure 7 presents the results at 150 kHz and 200 kHz, respectively for all the three wafers with drive voltage of 20 V, from which it can be found that the SH_0_ wave can be generated successfully for all three types of wafers. 

**Figure 8:**
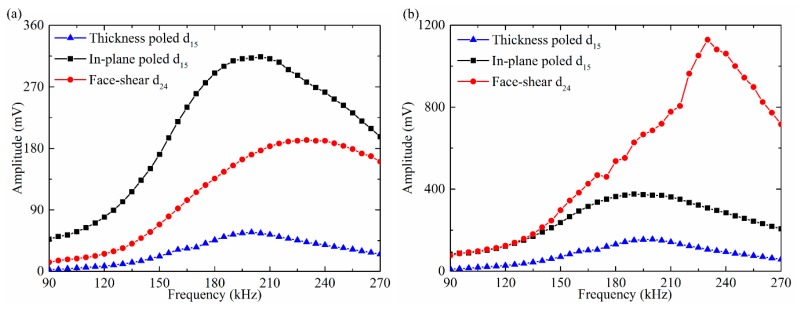
The amplitude of generated SH_0_ wave versus frequency. Signals were generated by different shear mode piezoelectric wafers and received by the d_36_ type PMN-PT wafer under (**a**) fixed drive voltage of 20 V and (**b**) fixed power consumption of 0.08 W.

The amplitude of the SH_0_ wave versus frequency was plotted in Figure 8. It can be seen from Figure 8a that for all the three types wafers, with the increasing frequency, the amplitudes of the generated SH_0_ wave increase first and then decrease gradually under the drive voltage of 20 V. Furthermore, the SH_0_ wave amplitude generated by the in-plane poled d_15_ wafer is always the maximum and that by the thickness-poled d_15_ wafer is always the minimum. The SH_0_ wave amplitude generated by the face-shear d_24_ wafer is always between the former two. Then, the three types shear mode wafers were excited by fixing the power consumption of 0.08 W, and the corresponding drive voltage for each wafer at different frequencies was calculated based on the Ohm’s law using the measured impedance spectrum in Figure 5. The obtained SH_0_ wave amplitude versus frequency was plotted in Figure 8b, which is quite different from Figure 8a. The thickness-poled d_15_ wafer still generates the minimum amplitude of SH_0_ wave. The face-shear d_24_ wafer generates SH_0_ wave with the same amplitude as that by the in-plane poled d_15_ wafer below 130 kHz. While above 130 kHz, the SH_0_ wave amplitude generated by the d_24_ wafer increased much quickly than that by the in-plane poled d_15_ wafer. These results indicated that the energy conversion efficiency of the face shear d_24_ wafer is better than that of the in-plane poled d_15_ wafer, and much better than that of the thickness-poled d_15_ wafer.

The testing results in Figure 8 can be explained as follows. When fixing the drive voltage, the energy consumption by the in-plane poled d_15_ wafer is the maximum because its impedance is the minimum, thus it always generated the maximum SH_0_ wave amplitude, as shown in Figure 8a. The other factor that can affect the generated wave amplitude is the deformation mechanism of the wafers. For the thickness shear d_15_ mode (both in-plane poled and thickness-poled), its deformation was actually the simple shear which was not self-balancing, which means the deformation cannot be transferred from the wafer to the waveguide in quasi-static case and can only be transferred in dynamic case via the inertial effect. This is why some researchers believe that the external constraint was needed for the thickness shear d_15_ wafers [34]. Figure 8b also showed that the wave driving efficiency of the in-plane poled d_15_ wafer was actually acceptable, even without any external constraint. In comparison, the face-shear deformation mode is self-balancing. Thus, the deformation could effectively be transferred to the hosting structure even in the quasi-static case without the inertial effect. Hence, when fixing the power consumption, the amplitude of SH_0_ wave generated by the face shear d_24_ wafer was maximum, as plotted in Figure 8b. The lowest amplitude of SH_0_ wave generated by the thickness-poled d_15_ wafer in Figure 8b indicated that although the thickness-poled d_15_ mode was equivalent to the in-plane poled d_15_ mode in deformation, its deformation which could propagate from the transducer to the waveguide was considerably lower.

### 4.2. Performances of Different Shear Mode Piezoelectric Wafers in SH Wave Reception

**Figure 9:**
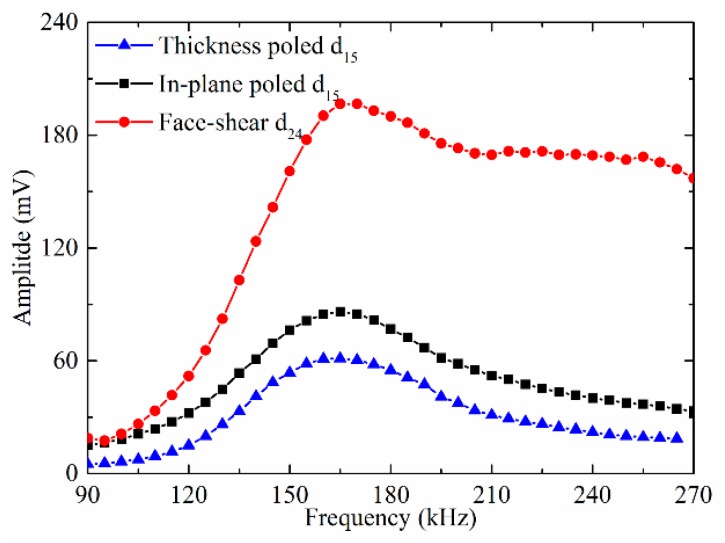
The amplitude of received SH_0_ wave versus frequency. Signals were generated by the d_36_ type PMN-PT wafer under 20 V and received by different shear mode piezoelectric wafers.

Next the performance of different shear mode piezoelectric wafers in SH_0_ wave reception was compared. The signals were generated by the d_36_ type PMN-PT wafer under 20 V and received by different shear mode piezoelectric wafers. The testing results were shown in Figure 9. It can be seen that within the testing frequency from 90 kHz to 270 kHz, the SH_0_ wave amplitude received by the face shear d_24_ wafer was always larger than that by the other two wafers. Above 160 kHz, the SH_0_ wave amplitude received by the face shear d_24_ wafer was about three times of that received by the in-plane poled d_15_ wafer and more than four times of that received by the thickness-poled d_15_ wafer. The excellent reception performance of the face-shear d_24_ wafer should be attributed to its in-plane shear deformation mechanism which is self-balancing. This result indicated that the face shear d_24_ wafer was much more suitable to act as an SH wave sensor than both types thickness shear d_15_ wafers.

### 4.3. Signals Generated and Received by the Same Shear Mode Piezoelectric Wafers 

**Figure 10:**
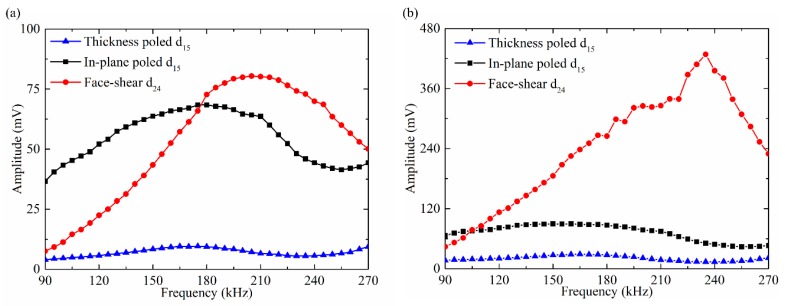
The SH_0_ wave amplitude versus frequency. Signals were generated and received by the same type shear mode piezoelectric wafer under (**a**) fixed drive voltage of 20 V, (**b**) fixed power consumption of 0.08 W.

In practical applications, wave signals are usually generated and received by the same type of transducers. Hence it is necessary to test wave signals generated and received by the same shear mode piezoelectric wafers. Figure 10a presented the results with the drive voltage of 20 V. It can be seen that in the whole testing frequency range from 90 kHz to 270 kHz, the SH_0_ wave amplitude generated and received by the thickness-poled d_15_ wafer was always the minimum. When the operating frequency was below 175 kHz, the SH_0_ wave amplitude generated and received by the in-plane poled d_15_ wafers was larger than that by the face shear d_24_ wafer. While above 175 kHz, the tendency was totally reversed.

When the PZT wafers were excited under the same power consumption, the results were quite different. As plotted in Figure 10b where all wafers were excited with the same power consumption of 0.08 W, the SH_0_ wave amplitude generated and received by the face shear d_24_ wafers was the maximum above 110 kHz. In most frequency ranges, its amplitude was over five times greater than that by the in-plane poled d_15_ wafer and more than ten times greater than that by the thickness-poled d_15_ wafer. These results were also in agreement with that plotted in Figure 8 and Figure 9. From these results, it can be inferred that the in-plane poled d_15_ wafer was more suitable to act as a SH wave transducer at low frequencies (below 175 kHz) but with large power consumption, while the face shear d_24_ wafer was more suitable to act as a SH wave transducer with high energy conversion efficiency within all the testing frequencies.

**Figure 11:**
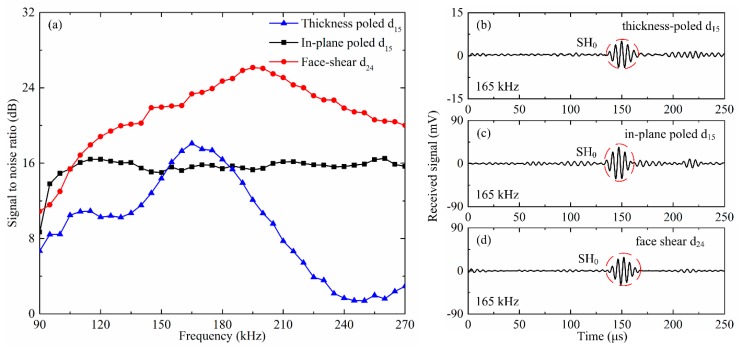
The signal to noise ratio (SNR) of SH_0_ wave generated and received by the same type shear mode piezoelectric wafers. (**a**) SNR verses frequency. Wave signals generated by (**b**) in-plane poled d_15_ wafer; (**c**) face shear d_24_ mode wafer and (**d**) thickness-poled d_15_ mode wafer under the drive voltage of 20 V at 165 kHz.

In practical applications, besides the wave amplitude, the signal to noise ratio (SNR) is another important parameter to evaluate a transducer. Thus, the SNR of SH_0_ wave generated and received by the same shear mode piezoelectric wafer with drive voltage of 20 V was plotted in Figure 11a. It can be found that the SNR of the SH_0_ wave generated/received by the face shear d_24_ wafer was more than 20 dB above 130 kHz, and can even reach 26 dB around 200 kHz. In comparison, the SNR of the SH_0_ wave by the in-plane poled d_15_ wafer was almost constant at around 16 dB above 110 kHz. The SNR of the SH_0_ wave by the thickness-poled d_15_ wafer was very small in most frequencies, while in a narrow band from 150 kHz to 180 kHz, it is slightly better than that by the in-plane poled d_15_ wafer, i.e., above 16 dB. 

To explain this, the wave signals generated by different types of wafers at 165 kHz were plotted in Figure 11b–d. It can be seen that the SNR for the face shear d_24_ wafer was maximum and that for the in-plane poled d_15_ wafer is minimum, which is in agreement with that in Figure 11a. Although the SH_0_ wave amplitude generated by the thickness-poled d_15_ wafer was much lower than that by the in-plane d_15_ wafer, its SNR was still slightly higher because of the much lower noise. It should be noted that the SNR of different types of wafers in SH_0_ wave generation is almost independent of the drive voltage. From the results in Figure 11, it can be concluded that the face shear d_24_ wafer was considerably better than the in-plane poled d_15_ wafer and much better than the thickness-poled d_15_ wafer in SH_0_ wave generation and reception.

Finally, in order to compare the performances of different shear mode piezoelectric wafers in a more intuitive manner, all the results presented above were summarized and listed in Table 1. It can be clearly seen that overall the face shear d_24_ wafer was considerably superior to the in-plane poled d_15_ wafers and much better than the thickness-poled d_15_ wafer when used as SH wave transducers.

## 5. Conclusions

In summary, we presented a systematic comparison of three types of shear mode piezoelectric wafers in SH wave generation and reception. The results indicated the face shear d_24_ wafer can generate almost single mode SH wave while the in-plane poled d_15_ wafer and the thickness-poled d_15_ wafer will generate both Lamb waves and SH wave. When served as an actuator, the in-plane poled d_15_ wafer can generate SH_0_ wave in high amplitude but with large power consumption, while the face shear d_24_ wafer can generate SH_0_ wave with high energy conversion efficiency. When served as a sensor, the face shear d_24_ wafer was obviously superior to the other two types of thickness shear d_15_ wafers. Besides, in most testing frequencies, the signal to noise ratio of the face shear d_24_ wafer was also the best in self-generation/reception. It should be mentioned that although the performances of the thickness-poled d_15_ wafer was not very good in SH_0_ wave generation and reception, it is very suitable for omni-directional SH wave piezoelectric transducers because of its unique configuration [27]. This work may provide useful guidance for SHM-based SH wave generation/reception.

## Figures and Tables

**Table 1:** The performances of different shear mode piezoelectric wafers in SH_0_ wave generation and reception.

Modes	Amplitude in Generation		Amplitude in Reception	Amplitude in Self-Generation/Reception		Signal to Noise Ratio (SNR)
	Fixed Voltage	Fixed Power Consumption		Fixed Voltage	Fixed Power Consumption	Fixed Voltage
In-plane poled d_15_	high	moderate	moderate	High below 175 kHz moderate above 175 kHz	moderate	~16 dB
Face shear d_24_	moderate	high	high	moderate below 175 kHz High above 175 kHz	high	typically above 20 dB
Thickness-poled d_15_	low	low	low	low	low	typically below 12 dB
